# Estimating the Risk of Parvovirus B19 Infection in Blood Donors and Pregnant Women in Japan

**DOI:** 10.1371/journal.pone.0092519

**Published:** 2014-03-21

**Authors:** Koji Nabae, Hiroshi Satoh, Hiroshi Nishiura, Keiko Tanaka-Taya, Nobuhiko Okabe, Kazunori Oishi, Kunichika Matsumoto, Tomonori Hasegawa

**Affiliations:** 1 Field Epidemiology Training Program, Infectious Disease Surveillance Centre, National Institute of Infectious Diseases, Tokyo, Japan; 2 Department of Social Medicine, Toho University School of Medicine, Tokyo, Japan; 3 Infectious Disease Surveillance Centre, National Institute of Infectious Diseases, Tokyo, Japan; 4 Graduate School of Medicine, The University of Tokyo, Tokyo, Japan; 5 Kawasaki City Institute of Public Health, Kanagawa, Japan; Technische Universität Dresden, Medical Faculty, Germany

## Abstract

**Background:**

Seroepidemiological study of parvovirus B19 has not taken place for some 20 years in Japan. To estimate the risk of parvovirus B19 infection in Japan among blood donors and pregnant women in this century, a seroepidemiological survey and statistical modeling of the force of infection were conducted.

**Methodology/Principal Findings:**

The time- and age-specific seroprevalence data were suggestive of strong age-dependency in the risk of infection. Employing a piecewise constant model, the highest forces of infection of 0.05 and 0.12 per year were observed among those aged 0–4 and 5–9 years, respectively, while estimates among older individuals were less than 0.01 per year. Analyzing the antigen detection data among blood donors, the age-specific proportion positive was highest among those aged 30–39 years, agreeing with the presence of dip in seroprevalence in this age-group. Among pregnant women, up to 107 fetal deaths and 21 hydrops fetalis were estimated to have occurred annually across Japan.

**Conclusions:**

Seroepidemiological profiles of PVB19 infection in Japan was characterized with particular emphasis on the risk of infection in blood donors and the burden of infection among pregnant women. When a vaccine becomes available in the future, a similar seroepidemiological study is expected to play a key role in planning the appropriate immunization policy.

## Introduction

Parvovirus B19 (PVB19) is one of the smallest viruses that are known to infect humans [1]. Since the virus was first reported in 1975, infection with PVB19 has been demonstrated to be associated with a variety of clinical manifestations. Among children, the most common clinical form of infection is erythema infectiosum (EI) which is also referred to as the slapped cheek syndrome or the fifth disease [2], [3]. EI is a relatively mild disease with non-specific influenza-like symptoms followed by facial rash which is considered to be caused by antibody-antigen immune complex depositions. Among adults, especially among middle-aged women, PVB19 infection can lead to clinically significant arthropathy. Moreover, among patients with increased erythropoiesis, PVB19 infection can cause transient aplastic crisis. Most importantly, PVB19 infection in a pregnant woman can lead to miscarriage or hydrops fetalis. Asymptomatic infection is seen in 25–50% of infections in host without comorbidity, and the estimated risk of transplacental infection among pregnant women is as high as 30% with a five to nine percent risk of fetal loss [4]. The transmission of PVB19 occurs primarily through droplets, but it can also be transmitted through blood products. A vaccine is presently under development [5].

While several industrialized countries regularly examine the epidemiological dynamics of PVB19 infection through laboratory (e.g. serological) investigations, Japan has been probably the only country in which epidemiological surveillance of EI has been conducted at a nationwide scale [6]. The number of clinically diagnosed EI cases has been continuously notified from approximately 3,000 pediatric sentinel sites across the country on a weekly basis since 1982. Surveillance data over the past 30 years has shown that epidemics of EI involve seasonality with a single annual peak in late June or early July and also a periodicity with four to six year cycles with geographic variations.

Published studies in other countries have indicated that seroprevalence of anti-PVB19 IgG increases with age: 2–15% among children below five years old, 15-60% among those aged 5–19 years and over 60% among adults [4], [7]–[11]. Those studies have also indicated that the circulation of PVB19 among children poses risks to adult groups, particularly among those aged 40 years and younger [12]. Since the potential risk of infection among blood donors and pregnant women represent two distinct social concerns over infection with PVB19, published seroepidemiological studies on PVB19 have estimated the risk among blood donors [4], [11] and investigated the burden of infection including fetal outcomes among pregnant women using seroepidemiological datasets and mathematical modeling techniques [7], [8], [12]. A modeling study in Europe has estimated the risk of PVB19 infection during pregnancy at 0.61% in Belgium, 0.69% in England and Wales, 1.24% in Finland, 0.92% in Italy, and 1.58% in Poland [12].

While seroepidemiological studies of PVB19 have also taken place in Japan [13], [14], they were conducted during 1970s-90s without an update for some 20 years, and moreover, epidemiological attempts have yet to explicitly estimate the risk of infection in blood donors and quantify the burden of infection among pregnant women. Using statistical modeling techniques, the present study aims to characterize the seroepidemiology of PVB19 infection in Japan, validating the prevalence estimate by looking into the antigen data among blood donors and estimating the risk of infection among pregnant women and fetal outcomes.

## Materials and Methods

### Seroepidemiological survey

The serum samples in the present study were derived from the National Epidemiological Surveillance of Vaccine-Preventable Diseases (NESVPD) [15] through which population-based seroepidemiological profiles have been regularly characterized for eight selected vaccine-preventable infectious diseases in Japan. This survey has taken place annually, collecting serum from at least 5,400 randomly sampled individuals across all age-groups. Participants are invited from randomly selected healthy individuals from whom survey officers were able to obtain informed consent. Such solicitation has taken place, for example, among local government officials and their families during routine health check-ups including those conducted among school children. There were no left-over samples of patients from hospitals and blood samples were obtained specifically for the purpose of this routine seroprevalence survey. Although PVB19 is not included in the selected eight vaccine-preventable diseases, we investigated a part of anonymized serum samples derived from two neighboring prefectures, Fukuoka and Saga, from 2004 to 2007, from the National Serum Reference Bank/Tokyo, National Institute of Infectious Disease, Japan, which stored the serum remnants of NESVPD.

To appropriately use the existing number of serum samples, we performed sample size calculations to determine the required number of samples. In advance of analyzing the serum samples, we examined a published seroprevalence study result in 1993 which had been conducted in three prefectures in Japan, including Fukuoka [14]. Assuming age-independence in the risk of infection with PVB19, the force of infection, *λ*, i.e., the rate at which susceptible individuals are infected, was estimated at 0.028 per year in 1993. Moreover, based on the census data in 2008, the average age of mothers for all births was estimated at 30.9 years old. Combining these two, it was implied that 1-exp(-30.9*λ*) = 58.3% of mothers are already immune by the age of 30.9 years, suggesting that 94 samples would be required to detect seroprevalence ±10% within a 95% confidence interval (CI). Considering differential fraction of immune individuals in other age-groups, we decided to examine 100 samples for each group, equally for 10 different age groups (0–4, 5–9, 10–14, 15–19, 20–25, 26–29, 30–35, 35–39, 40–49, and over 49 years). In total, 1000 samples from 2004-07 were investigated with a fixed male-female ratio at 1∶1 for each age-group and equal frequency for year of observation. To ensure data accuracy, avoiding under- or over-dilution, IgG antibody titer to PVB19 were examined in duplicate by enzyme immunoassay (EIA) using a commercial kit (Denka Seiken, Tokyo, Japan) according to the manufacturer's instructions. The ratio of the optical density for test specimen (average of two results for each specimen) to that of the control, hereafter referred to as the IgG antibody index, was calculated. If the antibody index was equal to or greater than 1.00, the test result was interpreted as positive. Samples that showed equivocal results at initial testing were retested. The seroepidemiological data in this study are available upon request from the corresponding author for noncommercial use.

### Statistical analysis and modeling

First, we examined the demographic characteristics of the obtained serum samples. In addition to gender- and age-specificities, we also investigated the presence of time-dependency during the sampling period from 2004-07. Since samples from different ages and years were taken from different individuals, we employed Welch analysis of variance (Welch ANOVA) and *χ* ^2^ test. The former test followed the test of normality (i.e., F test).

Second, the force of infection (i.e., the hazard rate of infection) was estimated in four different ways, i.e., (i) assuming time- and age-independence (i.e. a constant force of infection model), (ii) assuming age-dependence and employing a parametric model, (iii) assuming age-dependence and using a non-parametric model, and (iv) employing a time- and age-dependent model. The first three models used only datasets from 2004-07, but we additionally analyzed the 1993 data for model (iv) [14]. For now, we write the most explicit model with time- and age-dependence, because others are special cases of this type of force of infection model. Let *s*(*a*,*t*) be the proportion of susceptible individuals at age a and time *t*, the time- and age-dependent force of infection, *λ*(*a*,*t*), governs the dynamics as follows:
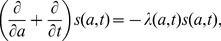
(1)with a boundary condition *s*(0,*t*) = 1 for any *t* (i.e. for simplicity, we ignore maternal antibody effect for the first six months of life). Integrating equation (1) along the characteristic line, we get
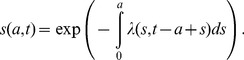
(2)


In the case of model (i), *λ* is a constant, and thus, the seroprevalence data are expected to be described by the equation 1-*s*(*a*) = 1-exp(-*λa*) at age *a* for cross sectional data. For models (ii) and (iii), only the time-element is dropped from (1) and the expected proportion of seropositive individuals, *i*(*a*), at age *a* is
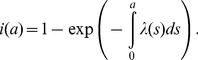
(3)


In the case of model (ii), we employed the well-known gamma-type parametric model as already proposed elsewhere [16]. For a non-parametric model (iii), we used a piecewise constant model with five unknown parameters, measuring forces of infection among those aged from 0–4, 5–9, 10–14, 15–39 years, and 40 years and older, following the discrete age-interval in a published study [7] and additionally separating adults into two groups by the common childbearing age of mothers (i.e. those aged 39 years and younger account for more than 96% of all births in Japan). For model (iv), we assumed for mathematical convenience that time- and age-elements are separable, i.e., *λ*(*a*,*t*) =  *λ*_a_(*a*)*λ*_t_(*t*), and employed exactly the same piecewise constant model for the age-dependent part as was assumed in model (iii). The time-specific forcing, *λ*_t_(*t*), was also dealt with as a piecewise constant model with five unknown parameters, i.e. 1972 and earlier, 1973–82, 1983–92, 1993–2002, and 2003 and later. Maximum likelihood estimates of parameters were obtained by minimizing the negative log-likelihood that rested on binomial deviance as described elsewhere [16], [17]. The 95% CI was derived from the profile likelihood. Goodness-of-fit of models (i) – (iii) were compared with each other using the Akaike's Information Criterion (AIC) [18]. The last model (iv) used additional seroprevalence data in 1993, and moreover, the estimated time-dependent forcing was overlaid with the notification data of EI from the abovementioned two prefectures based on sentinel surveillance from 1982.

As a measure of transmissibility, the basic reproduction number, *R*_0_, was computed. When using the constant force of infection in model (i), we employed a homogeneous mixing assumption and assumed that the average life expectancy at birth is *L* = 80 years with a rectangular shape survivorship, so that we have *R*_0_ = *λL*. We also used the age-dependent force of infection from model (iii) to estimate *R*_0_ employing the following estimator derived by Farrington et al. [19]: 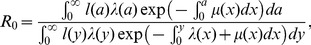
(4)where *l*(*a*) is the leading left eigenfunction of age-dependent transmission rate. *μ*(*a*) is the age-specific mortality rate, and for consistency, we again employed the rectangular shape survivorship. Since the piecewise constant model is discrete, we derived the following discrete version of the estimate of *R*_0_:
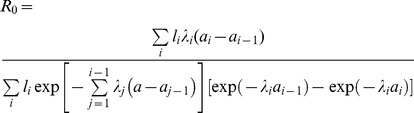
(5)where *a*_i_ represents the upper age bound of age-group *i*. The left eigenfunction, *l*_i_ of the contact matrix was derived from published survey data in the United Kingdom [20] with an adjustment of age-specific population size to Japanese data, assuming that age-specific contact pattern in Japan is the same as that in the United Kingdom [21], [22]. Because it is difficult to estimate the sampling distribution of *R*_0_, the 95% percentile confidence intervals were obtained by employing a bootstrapping method [23].

### Risk estimation in blood donors and pregnant women

Using the age-specific proportion of antigen positives among blood donors, we calculated the age-specific risk of PVB19 infection, directly from empirical data. The dataset of antigen testing results was available from 2001 to 2007, and during this period, a receptor-mediated hemagglutination (RHA) assay was used for screening. Age distribution of blood donors was extracted from the latest available statistics of the Japanese Red Cross Society [24]. Assuming that the risk of positive blood sample is binomially distributed, we obtained the 95% CI of prevalence. We also obtained data on the age-specific proportion of IgM antibody positive individuals against PVB19 from February 2008 to January 2009, as a marker of recent PVB19 infection. A total of randomly selected 651 blood donors were tested for IgM using EIA at the Japanese Red Cross Osaka Blood Center.

Subsequently, we estimated the burden of PVB19 infection among females at childbearing age including the estimated number of infections during pregnancy in Japan. For the calculation, we followed published studies in the United Kingdom [7], [8], adopting a random sampling assumption of pregnant women from all women in an identical age-group. Since all pregnancy events have not been stratified by the age of pregnant women in the Japanese census record, we used the reported number of birth events as an approximate of all pregnancy events. We used the confidence intervals of the age-specific force of infection based on a piecewise constant model (iii) and adopted the published risk estimates of fetal death and hydrops fetalis due to maternal infection during the first 20 weeks of pregnancy for the former and from weeks 9 to 20 for the latter at 9.0% and 2.9%, respectively [25], as already practiced elsewhere [8]. We did not use estimates of the force of infection from the time- and age-dependent model (iv) for abovementioned calculations because the 95% CIs of age-dependent element were only partially calculable due to limited sample size. All statistical data were analyzed using the statistical software JMP ver. 9.0.0 (SAS Institute Inc., Cary, NC). The study protocol was reviewed and approved by the Institutional Review Board at the Toho University School of Medicine.

## Results

### Descriptive seroepidemiology

**Figure 1:**
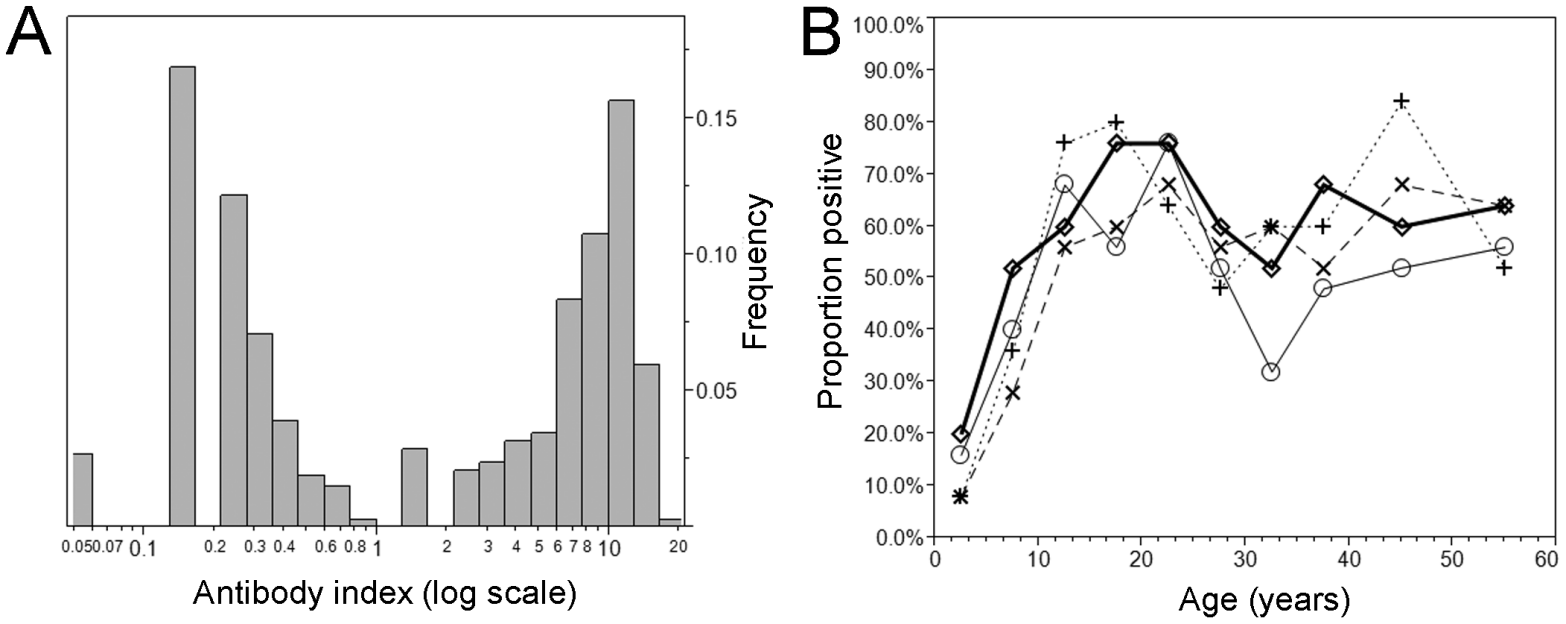
Distribution of the IgG antibody index and the time- and age-dependent proportion seropositive against parvovirus B19 in Japan from 2004-07. A. The distribution of IgG antibody index against parvovirus B19. The antibody index was calculated as the ratio of the optical density of test sample to that of control. Using a logarithmic scale for the horizontal axis, a bimodal shape is clearly identified. B. The time- and age-dependent proportion of seropositive against parvovirus B19. The collection of serum samples took place in 2004 (unfilled circles with thin solid line), 2005 (crosses with dotted line), 2006 (diamonds with thick solid line) and 2007 (x marks with dashed line), respectively.

Of 1,000 serum samples tested in duplicates, eight samples yielded equivocal results, and thus, the eight were re-tested in duplicates, allowing six results to yield agreed result (and only two remained to be equivocal with the antibody index ranging from 0.8–1.0). Figure 1A shows the distribution of the antibody index. Taking a logarithmic scale for the antibody index axis, two distinct peaks were identified. As suggested for the interpretation of the testing result, the bimodal distribution was confirmed to be clearly separated at the cut-off value of 1.00. In total, 543 individuals tested positive (54.3%). Figure 1B shows the age-specific proportion seropositive by sampling year. Mean age (and the standard deviation) of seropositive individuals was 28.1 (14.8) years, while seronegative individuals were significantly younger with mean age of 22.4 (17.1) years (p<0.01; Welch ANOVA). As indicated by overlaps of seropositive fraction for multiple times in Figure 1B (i.e. multiple crossing points between two different survival curves), no significant difference was identified by the year of sampling, and thus, the subsequent analysis used the aggregated data for all 4 years to quantify the transmission dynamics. There was no significant gender specificity in seroprevalence (p = 0.48; χ^2^ test).

### Force of infection and age-specific seroprevalence

Assuming an age-independent risk of infection, the force of infection is estimated at 0.031 per year (95% CI: 0.029, 0.034) which was not significantly deviated from the estimate from 1993 data (the 95% CI ranged from 0.025 to 0.031 per year). Assuming a rectangular age distribution, the basic reproduction number, *R*_0_, based on the constant force of infection model, was estimated as 2.51 (95% CI: 2.30, 2.74). Moreover, the constant force of infection model indicated that the average age at infection is 1/*λ* = 31.8 years old (given the absence of age-dependence in the risk of infection).

**Figure 2:**
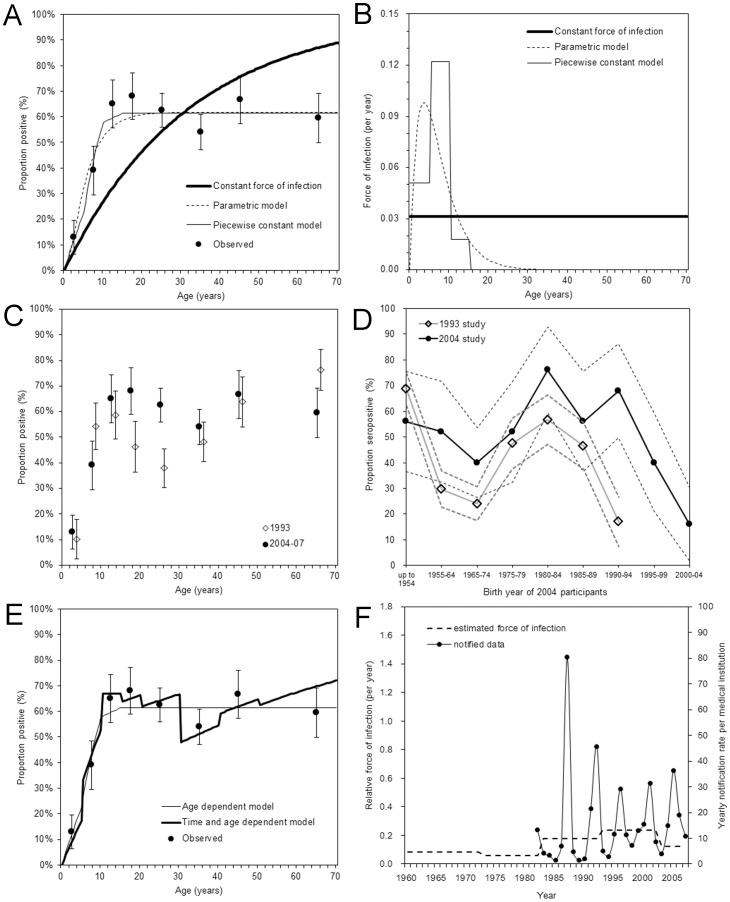
Seroepidemiological investigation of parvovirus B19 infection in Japan. A. Comparison between observed and predicted proportion of seropositive from 2004–07 without accounting for time-dependency. The mean of observed data are shown as filled circles with the 95% confidence intervals by whiskers. Thick solid line represents the prediction based on a constant force of infection model, dashed line a gamma-type age-dependent model, and thin solid line a piecewise constant age-dependent model. B. Estimated force of infection with parvovirus B19 in Japan. The differential styles of lines correspond to those in panel A. C. Comparison of observed seroprevalence data between 1993 and 2004–07. Filled circles represent the observed data from 2004–07, while unfilled diamonds represent the data from 1993. Whiskers extend to both ends of the 95% confidence intervals. D. Comparison of seroprevalence data between 1993 and 2004 by birth year. Dashed lines represent the 95% confidence intervals. The horizontal line shows the birth year of 2004 participants, while the birth year of 1993 participants is calculated as the birth year minus 1 (i.e. those born from 1955–64 for 2004 participants are compared against those born from 1954–63 for 1993 participants). E. Comparison of predicted data from 2004–07 using age-dependent model and age- and time-dependent model. Filled circles represent the observed data, and whiskers extend to both ends of the 95% confidence intervals. Thick line shows the prediction based on time- and age-dependent assumption. F. The comparison between the time-dependent element of the force of infection (dashed line; left vertical axis) and the annual notification rate of erythema infectiosum from sentinel medical institutions in Fukuoka and Saga prefectures, Japan, from 1982 onwards (circles and solid line; right vertical axis).

However, the goodness-of-fit for two other models were better than that of the constant force of infection model (Figure 2A). Employing the age-dependent force of infection either by the parametric or the non-parametric model, all expected values were included within the 95% CI of the observed proportion of seropositive. AIC values for models (i), (ii) and (iii) were 203, 60.1 and 60.3, respectively, indicating that models (ii) and (iii) were almost equally good and much better than the model (i) in describing the observed pattern of the data. Figure 2B compares the estimated force of infection by three different models. In the case of the age-dependent parametric model (ii), the peak of infection was expected to occur at around the age of 5 years, while the piecewise constant model (model (iii)) predicted that the highest force of infection 0.12 (95% CI: 0.00, 0.21) per year was seen among those aged 5–9 years and the second highest force of infection 0.05 (95% CI: 0.02, 0.10) among those aged 0–4 years. For model (iii), upper bounds of the force of infection among those aged 15–39 years and 40 years and older were 0.0059 and 0.0246 per year, respectively. The *R*_0_ using the age-dependent piecewise constant model was estimated to be 2.07 (95% CI: 1.33, 2.98), the expected value of which was smaller than the estimate based on the constant force of infection model, but the difference was not significant.

Figure 2C compares the observed age-specific proportions of seropositive between 1993 and 2004-07, both of which were used to parameterize the time- and age-dependent model (iv). Although no significant difference can be identified in the constant force of infection between 1993 and from 2004-07, we specifically employed model (iv) to describe the time-dependent shift in a dip in seropositive proportion from those aged 20-29 years in 1993 to 30–39 years from 2004-07. Comparing seroprevalence by birth year (Figure 2D), it can be seen that the dip in seroprevalence is observed in a birth cohort born from 1965-74. The relative frequency of age-dependent forces was similar to the age-only model (iii) in Figure 2B. The age-element of model (iv) was compared against the observed seroprevalence data from 2004-07 (Figure 2E) and the time-dependent element was compared against the notification data in Figure 2F. The average over the time-interval was only crudely captured (i.e. as was indicated by estimates, there was no dramatic time-dependent trend in the risk of infection), and a sharp peak in 1987 was perhaps smoothed out by adjacent years and was not reflected in the estimated force of infection from 1983-92. Although the time-element was thus not strongly aligned with the notification data, the predicted data allowed us to realize the dip in seroprevalence among those aged in their 30s (Figure 2E).

### Blood donors

**Figure 3:**
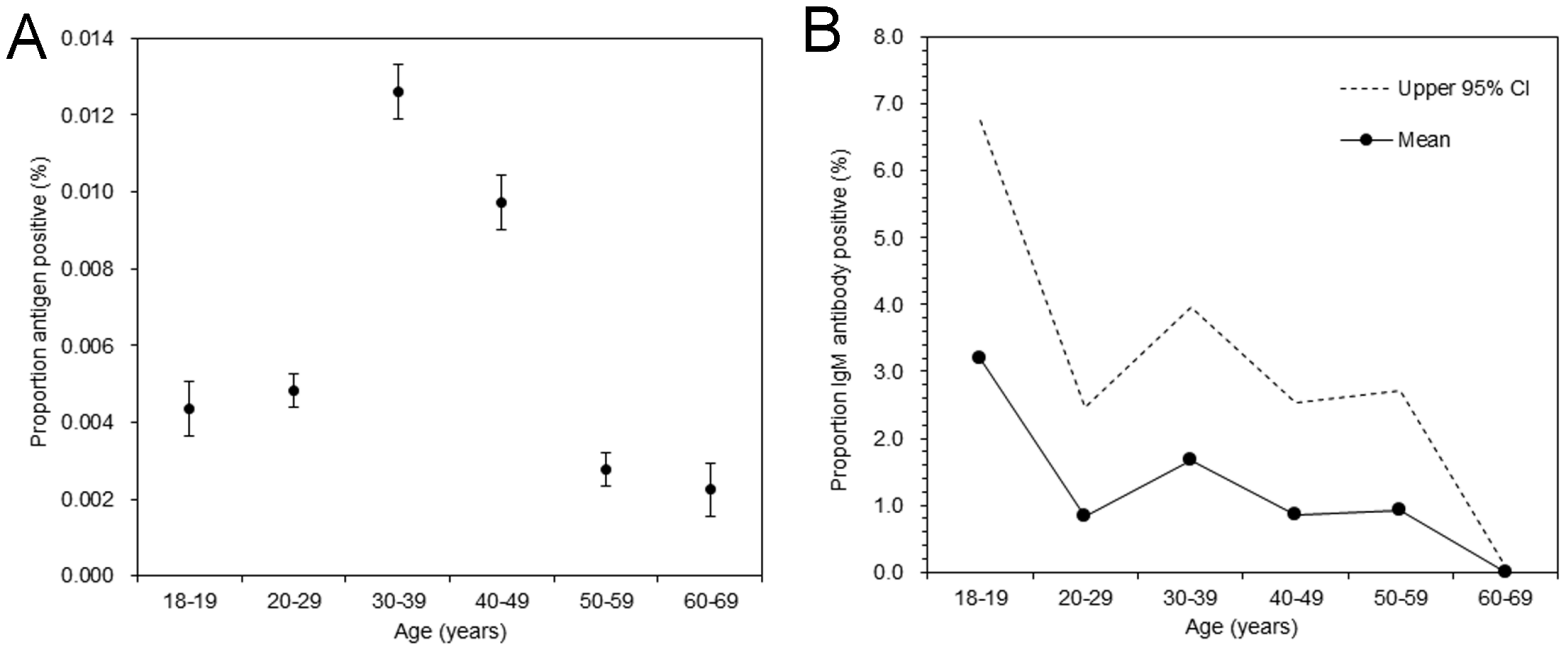
Age-specific proportions positive for antigen and IgM antibody against parvovirus B19 among blood donors from 2001–2007 in Japan. A. Observed proportion of antigen positive as a function of age. Filled circles represent the mean, and whiskers extend to both ends of the 95% confidence intervals. A receptor-mediated hemagglutination (RHA) assay was used. B. Observed proportion of IgM antibody positive. Solid line and filled circles represent the mean, with the upper 95% confidence interval given by dashed line. Lower bound is identical to the horizontal axis.

Assuming a random sampling assumption of blood donors from the entire population, the age-specific proportion of antigen positive was calculated among blood donors as a possible direct measurement of the risk of infection. From 2001-07, a total of 38 million persons donated blood, among which 2,806 tested positive for antigen (prevalence: 7.4 (95% CI: 7.1, 7.7) per 100,000 donors). Figure 3A shows the age-specific proportion of antigen positive. Those aged 30–39 years yielded the highest prevalence and those aged 40–49 yielded the second highest estimate. Compared to those aged 20–29 years, the prevalence among those aged 30–39 and 40–49 years were 2.6 and 2.0 times higher, respectively. Figure 3B shows the age-specific proportion of IgM antibody positive. Of 651 samples, 8 tested positive (1.2% (95% CI: 0.4, 2.1)), and thus, the uncertainty was large, but the proportion positive declined almost monotonically with age.

### Pregnant women

Table 1 shows the estimated age-specific numbers of infection among women at child-bearing age and during pregnancy, along with rough corresponding estimates of fetal deaths and hydrops fetalis. Although the precision is limited in the estimates, up to 2374 infections are estimated to occur annually among pregnant women in Japan. Assuming that the risk of fetal death following PVB19 infection during the first 20 weeks of pregnancy is 9.0%, up to 107 fetal deaths are anticipated per year. Similarly, adopting 2.9% as the risk of hydrops fetalis among pregnant women at weeks 9–20, up to 21 cases are estimated annually.

**Table 1:** Estimated age-specific annual numbers of parvovirus B19 infections and associated complications among women at child-bearing age in Japan.

Age group (years)	No. females	No. maternities	Total infections	Infections during pregnancy	Fetal deaths	Hydrops fetalis
15–19	2,958,000	13,273	156 (0–6,585)	1 (0–30)	0 (0–1)	0 (0–1)
20–24	3,116,000	104,053	164 (0–6,736)	5 (0–225)	0 (0–10)	0 (0–2)
25–29	3,546,000	300,350	187 (0–7,445)	16 (0–631)	1 (0–28)	0 (0–5)
30–34	3,987,000	373,452	210 (0–8,129)	20 (0–761)	1 (0–34)	0 (0–7)
35–39	4,785,000	221,245	251 (0–9,475)	12 (0–438)	1 (0–20)	0 (0–4)
40–44	4,609,000	37,435	8 (0–35,597)	0 (0–289)	0 (0–13)	0 (0–3)
Total	23,001,000	1,049,808	976 (0–73,967)	54 (0–2,374)	3 (0–107)	0 (0–21)

Maximum likelihood estimates with the 95% confidence intervals (inside parentheses) are shown for the right four columns. The 95% confidence intervals for the estimated force of infection based on a piecewise constant model were used: i.e., 0–0.0059 for those aged 15–39 years and 0–0.0246 for those aged 40 years and older. Risks of fetal death from gestational week 1–20 and hydrops fetalis from week 9–20 were assumed to be 9.0% and 2.9%, respectively [8], [25].

## Discussion

The present study investigated the seroepidemiological profiles of parvovirus B19 infection in Japan. Whereas various clinical studies had taken place in Japan in advance of the present study including those focused on pregnant women [26], [27], to the best of our knowledge, the present study is the first to explicitly estimate the frequency of infection in blood donors and the burden of infection among pregnant women in this country. The estimated measure of the transmissibility, *R*_0_, was 1.3–3.0, which did not deviate from an earlier estimate ranging from 2.6–3.5 based on seroprevalence survey in the Netherlands [4]. Across Japan, it was estimated that up to 2374 infections could have occurred during pregnancy, although the uncertainty bound was wide, ranging from 0 to 2374 (as was also the case in the UK study [8]). Similar estimates of the force of infection between Europe and Japan indicate that the level of endemicity for PVB19 (i.e. frequency of infection) and the contact pattern are likely similar to each other.

Two specific lessons should be learnt from our exercise. First, a strong age-dependency in the force of infection was observed, while no obvious indication of time-dependent change (e.g. declining trend) in the proportion of seropositive was seen from 1993 to 2007. The highest frequency of infection was seen among those aged below 10 years, and thereafter both parametric and non-parametric models agreed that the annual risk of infection was at most 1%. That is, the transmission dynamics of PVB19 is likely regulated by and maintained among children, yielding very important implications for future control planning including age-dependent vaccination strategy. In fact, the risk of infection among pregnant women is known to be higher in households with small children than those without [10], [12] (which could partly explain the observed peak among those in their 30s in Figure 3A), and thus, within household contact between pregnant women and children could play a key role in determining the optimality of controlling the transmission by targeting children [28]. The uncertainty in the age-dependent contact patterns could be a plausible explanation for observing differential risk of infection among pregnant women across European countries [12]. In the future, the age-dependency should be closely monitored even in the absence of vaccination, because a shift (e.g. delay) in the age at infection can vary (e.g. increase) the number of infections among pregnant women, as was observed for rubella under a partial vaccination [29], [30].

Second, in addition to age-dependent estimates of the force of infection that could measure the incidence of infection among blood donors (Figure 2B), we also analyzed the antigen screening results among blood donors (Figure 3A). Age-specific proportion of antigen positive yielded the peak among those aged 30–39 years followed by 40–49 years. A similar age-specific antigen pattern was previously reported from the Netherlands [31], but no explicit reason has been clarified for this observation. The peak in prevalence among those in their 30s agreed well with the dip in age-specific seroprevalence, possibly reflecting the absence of major epidemics during childhood among those born during 1965-74 and substantial number of transmission events from children to parents. Nevertheless, the antigen positive estimates among those in their 30s and 40s were more than double of those in their 20s (while the fraction susceptible, based on seroprevalence survey, was not as different as the proportion of antigen positive).Moreover, IgM antibody data yielded an approximately monotonic decline in the age-specific proportion positive. These findings were not fully consistent with anticipating substantial child-to-parent transmissions in explaining the observed pattern, and other reasons might also explain observed phenomena (e.g. other factors, including age-dependent biological reaction to the virus and possible sampling effect (i.e. blood donors may not have well represented the general population), might also explain the observed pattern).

Four limitations should be noted. First, our subjects were limited to the population in Fukuoka and Saga prefectures, both of which are located on Kyushu Island, the western part of Japan. Our estimates involve a limitation in the representativeness of the finding and may not fully reflect that of entire Japan. Nevertheless, rather than ensuring the representativeness, the present study focused on two prefectures as the first attempt to explicitly characterize infection risks while allowing comparability between 1993 data and the result from 2004-07. Second, the estimated risk of infection among blood donors (Figure 3A) and pregnant women (Table 1) rested on an assumption that they were randomly sampled from the population irrespective of infection status. However, as briefly noted above, household structure (e.g. if a pregnant woman has any children) and other risk factors are known to influence the risk of infection with PVB19 during pregnancy. A more precise estimation would require us to account for known epidemiological risk factors using a more sophisticated statistical model. Third, we have not considered the seasonality and periodicity in the model; however, the sample size was limited and the fluctuation is known to be smoothed out over a long time yielding only marginal impact on the estimate of the transmissibility [32]. Lastly, small errors in antigenic testing results, e.g. non-specific false positive results and involvement of repeaters of blood donation due to a long viremic period without symptoms, cannot be avoided in the empirical observation.

Despite these limitations, the present study characterized seroepidemiological profiles of PVB19 infection in Japan, with particular emphasis on the risk of infection in blood donors and the burden of infection among pregnant women. When a vaccine becomes available in the future, a similar seroepidemiological study is expected to play a key role in determining appropriate immunization policy. We believe that the present study contributed to clarifying the key element of the epidemiology of PVB19 in Japan.
